# Prediction models for differentiating benign from malignant liver lesions based on multiparametric dual-energy non-contrast CT

**DOI:** 10.1007/s00330-024-11024-8

**Published:** 2024-08-26

**Authors:** Takashi Ota, Hiromitsu Onishi, Hideyuki Fukui, Takahiro Tsuboyama, Atsushi Nakamoto, Toru Honda, Shohei Matsumoto, Mitsuaki Tatsumi, Noriyuki Tomiyama

**Affiliations:** 1 Department of Diagnostic and Interventional Radiology, Osaka University Graduate School of Medicine, Osaka, Japan; 2 Department of Medical Physics and Engineering, Osaka University Graduate School of Medicine, Osaka, Japan

**Keywords:** Liver neoplasms, Logistic regression, Logistic models, Differential diagnosis, X-ray computed tomography

## Abstract

**Objectives:**

To create prediction models (PMs) for distinguishing between benign and malignant liver lesions using quantitative data from dual-energy CT (DECT) without contrast agents.

**Materials and methods:**

This retrospective study included patients with liver lesions who underwent DECT, including non-contrast-enhanced scans. Benign lesions included hepatic hemangioma, whereas malignant lesions included hepatocellular carcinoma, metastatic liver cancer, and intrahepatic cholangiocellular carcinoma. Patients were divided into derivation and validation groups. In the derivation group, two radiologists calculated ten multiparametric data using univariate and multivariate logistic regression to generate PMs. In the validation group, two additional radiologists measured the parameters to assess the diagnostic performance of PMs.

**Results:**

The study included 121 consecutive patients (mean age 67.4 ± 13.8 years, 80 males), with 97 in the derivation group (25 benign and 72 malignant) and 24 in the validation group (7 benign and 17 malignant). Oversampling increased the benign lesion sample to 75, equalizing the malignant group for building PMs. All parameters were statistically significant in univariate analysis (all *p* < 0.05), leading to the creation of five PMs in multivariate analysis. The area under the curve for the five PMs of two observers was as follows: PM1 (slope *K*, blood) = 0.76, 0.74; PM2 (slope *K*, fat) = 0.55, 0.51; PM3 (effective-*Z* difference, blood) = 0.75, 0.72; PM4 (slope *K*, blood, fat) = 0.82, 0.78; and PM5 (slope *K*, effective-*Z* difference, blood) = 0.90, 0.87. PM5 yielded the best diagnostic performance.

**Conclusion:**

Multiparametric non-contrast-enhanced DECT is a highly effective method for distinguishing between liver lesions.

**Clinical relevance statement:**

The utilization of non-contrast-enhanced DECT is extremely useful for distinguishing between benign and malignant liver lesions. This approach enables physicians to plan better treatment strategies, alleviating concerns associated with contrast allergy, contrast-induced nephropathy, radiation exposure, and excessive medical expenses.

**Key Points:**

- *Distinguishing benign from malignant liver lesions with non-contrast-enhanced CT would be desirable*.
- *This model, incorporating slope K, effective Z, and blood quantification, distinguished benign from malignant liver lesions*.
- *Non-contrast-enhanced DECT has benefits, particularly in patients with an iodine allergy, renal failure, or asthma*.

**Graphical Abstract:**

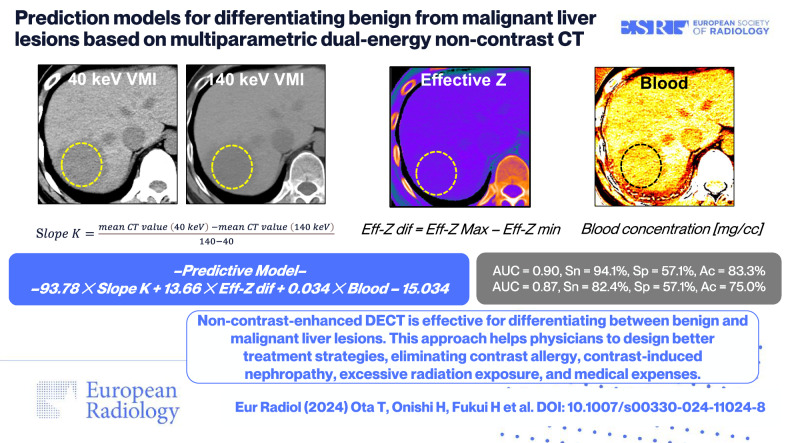

## Introduction

Liver CT allows accurate differentiation of various focal liver lesions and usually requires the administration of contrast media [1]. Considering the adverse side effects of contrast media, including contrast-induced nephropathy, and medical economics, it would be advantageous to evaluate liver lesions using CT without contrast media [2]. Although most side effects to nonionic iodinated contrast media are often mild and temporary, severe and life-threatening side effects may even result in death [3]. However, distinguishing liver lesions on non-contrast-enhanced CT (non-CECT) is challenging in clinical practice. The clinical question that needs to be answered is how effectively non-CECT can distinguish liver lesions using the imaging techniques available today.

To date, researchers have evaluated liver lesions using non-CECT images by employing techniques such as radiomics and texture analysis. These techniques have demonstrated a certain degree of capability in differentiating between different liver lesion types [4–7].

Dual-energy CT (DECT) has seen significant developments and has recently become a prevalent and extensively used technique in routine clinical practice [8]. DECT employs dual kilovoltage acquisitions of identical scan volumes to enable material differentiation. Spectral or multi-energy CT also referred to as DECT, offers additional image reconstructions to enhance the precision of CT analysis. DECT, a commonly employed method in clinical practice, enables the generation of virtual monochrome images (VMI) at various energy levels [8]. DECT can utilize material decomposition (MD) techniques to obtain mass density, effective atomic number, and other material-specific information for different analyses [8]. Although DECT is recognized for its ability to provide extensive imaging data, its potential application for differentiating liver lesions from non-CECT remains unexplored.

Our hypothesis is that the use of various quantitative data obtained from non-contrast-enhanced DECT (nCE-DECT) could facilitate the differentiation of liver lesions. Hepatic hemangioma (HH) is the most common type of benign liver lesion in clinical practice [9]. Hepatocellular carcinoma (HCC), metastatic liver cancer (MLC), and intrahepatic cholangiocellular carcinoma (ICC) are the most common types of malignancies, comprising most cases [10, 11]. Therefore, we endeavored to incorporate these hepatic lesions into our assessment. The objective of this study was to develop prediction models (PMs) for differentiating between benign liver lesions (HH) and malignant liver lesions (HCC, MLC, and ICC) using multiple quantitative parameters from nCE-DECT.

## Materials and methods

### Demographics of the patients

**Fig. 1:**
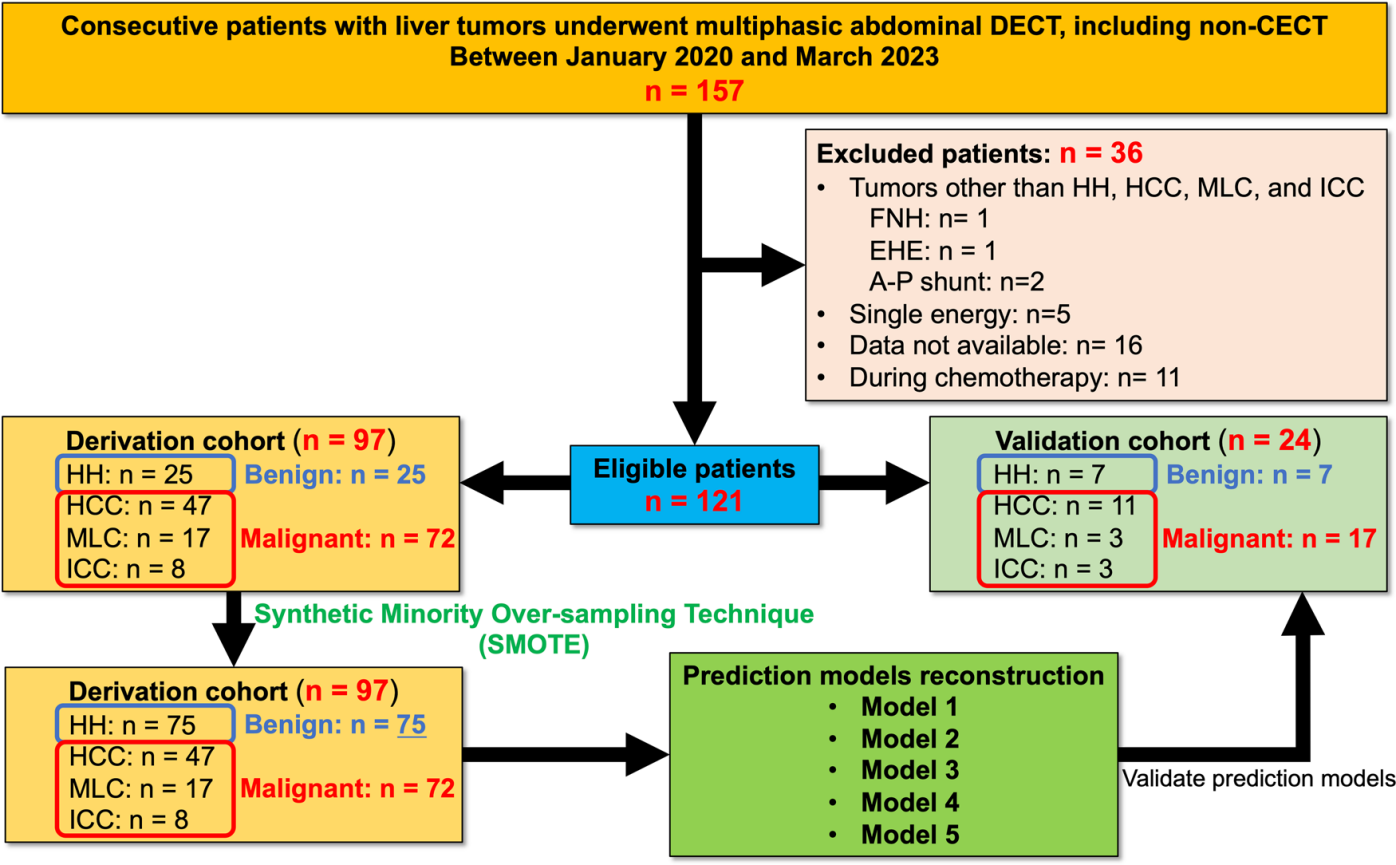
Enrollment of patients

The institutional review board and local ethics committee approved this university medical center retrospective study. Because this research was retrospective and non-interventional, informed consent was waived. The study included patients with liver lesions who underwent multiphasic abdominal DECT, including non-CECT, from January 2020 to March 2023. Patient exclusions included (1) liver lesions other than HH, HCC, MLC, and ICC; (2) single-energy mode scanning; (3) technical difficulties when DECT data were unavailable; and (4) patients during chemotherapy. The final study population included the derivation and validation cohorts. The derivation and validation cohorts were randomly assigned 80:20 (Fig. 1). In addition, the prevalence of liver cirrhosis was evaluated in our cohort. Liver cirrhosis was defined as follows.

1. Histopathological criteria: with respect to the New Inuyama Classification, liver cirrhosis is defined as F4 (bridging fibrosis with nodule formation).

2. Clinical criteria: suggestive liver cirrhosis included physical signs such as spider angiomas, palmar erythema, ascites, and splenomegaly; blood test abnormalities such as thrombocytopenia, hypoalbuminemia, and prolonged prothrombin time; and imaging features such as liver surface irregularity, atrophy, splenomegaly, and collateral circulation development [12].

### Diagnostic criteria for liver lesions on CT and MRI

We employed the following diagnostic criteria based on CT and MRI for liver lesions without a histopathological diagnosis:

1. HH: a well-circumscribed lesion exhibiting peripheral nodular enhancement and progressive centripetal fill-in on multiphasic CECT and MRI. Hyperintense and a “light-bulb” bright signal on T2-weighted images [13].

2. HCC: non-rim arterial phase hyperenhancement, non-peripheral washout in the portal venous and equilibrium phases, and the presence of an enhancing “capsule” on multiphasic CECT and MRI [14].

3. MLC: lesions that exhibit either hypervascular or hypovascular patterns, depending on the type of primary lesions. Peripheral ring-like enhancement and central necrosis on CECT and MRI. Extrahepatic disease history may aid in the diagnostic process [15].

4. ICC: a mass-forming lesion characterized by irregular peripheral enhancement during the arterial phase and delayed enhancement due to fibrosis. In addition, vascular encasement, capsular retraction, and segmental biliary dilatation may be observed [13].

### Collection of DECT data

All nCE-DECT images were acquired using a 256-channel multidetector CT scanner (Revolution CT; GE HealthCare). The GSI assist feature controls exposure automatically. The scan parameters were 80/140 kVp tube voltage with fast switching and 5 mm image thickness. The tube current, rotation time, and helical pitch are automatically adapted to ensure image quality comparable to a 13 noise index. Medium-strength deep learning image reconstruction (True Fidelity, GE HealthCare) was used to reconstruct nCE-DECT images. The standard reconstruction kernel was used to reconstruct all nCE-DECT images with a matrix size of 512 × 512.

### Quantitative analysis of nCE-DECT images

**Fig. 2:**
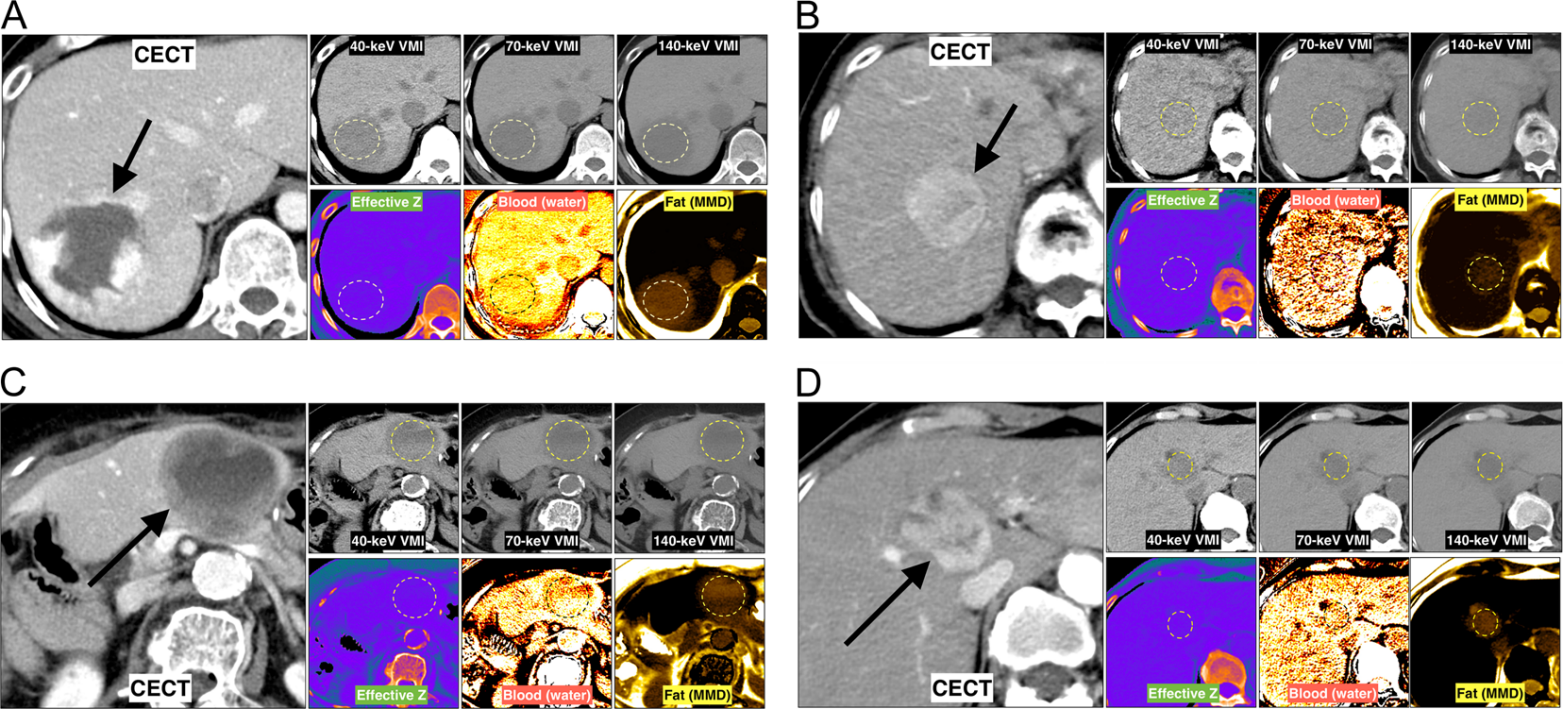
Images of virtual monoenergetic and MD derived from nCE-DECT in representative cases of liver lesions. **A** A 46-year-old male was diagnosed with HH. A mass measuring 60 mm in diameter with peripheral nodular enhancement is observed in the liver S7 on the contrast-enhanced CT (CECT) of the portal venous phase. Additional images, including 40 keV, 70 keV, and 140 keV virtual monoenergetic images (VMI), effective *Z* images, quantitative images of blood using water as the material pair, and quantitative images of fat using multi-material decomposition (MMD), were generated from the nCE-DECT. In this case, the diagnosis of HH was determined using typical contrast-enhanced CT imaging findings. **B** A 91-year-old woman was diagnosed with HCC. The arterial phase CECT reveals a 35-mm-diameter mass in S8 of the liver that exhibits arterial hyperenhancement. Images in the top row were acquired with nCE-DECT at energy levels of 40 keV, 70 keV, and 140 keV. Images in the lower row demonstrate the effective *Z*, blood (water), and fat (MMD) content. The diagnosis of HCC was determined based on imaging evidence from contrast-enhanced CT, contrast-enhanced MRI, and contrast-enhanced US. Heavy particle radiotherapy was administered in this case. **C** An 80-year-old woman with a hepatic metastasis originating from a gastrointestinal stromal tumor (GIST). By means of CECT of the portal venous phase, a ring-enhanced mass with a diameter of 58 mm is observed in the lateral segment of the liver. The displayed images are those produced by nCE-DECT at 40 keV, 70 keV, and 140 keV VMI, Eff-*Z*, blood (water), and fat (MMD). In this case, a surgical operation was conducted, and three pathologists diagnosed liver metastases from GIST histologically. **D** A 68-year-old woman was diagnosed with ICC. An arterial phase CECT scan reveals a 37-mm-diameter mass with heterogeneous enhancement in the liver S4. Images obtained using 40 keV, 70 keV, and 140 keV VMI, Eff-*Z*, blood (water), and fat (MMD) images generated via nCE-DECT are displayed. A surgical operation was conducted, and three pathologists diagnosed the presence of mixed-type HCC and ICC histologically

The nCE-DECT images were transferred to an Advantage Workstation 4.5 (AW 4.5; GE HealthCare). In a derivation cohort, two abdominal radiologists, Observers 1 and 2, with 11 years of experience, objectively analyzed nCE-DECT data with a 5-mm slice thickness using a GSI Viewer (GE HealthCare). Two independent observers (Observers 1 and 2) calculated the average CT number of liver lesions with elliptical to circular ROIs at VMIs of 40 keV, 70 keV, and 140 keV. When multiple liver lesions were present, only the largest were examined. The reason for choosing the largest lesions was that small lesions are difficult to identify on non-CECT, and it is difficult to place ROIs. To develop predictive models using non-CECT, the ROI was positioned as homogeneously as possible in the region, with particular attention paid to avoiding necrosis or degeneration areas that were obviously apparent on non-CECT (Fig. 2). Internal necrosis in MLC frequently exhibits a relatively homogeneous low-density in non-CECT images; therefore, we placed the ROI throughout the homogeneous low-density area (Fig. 2C). Although the necrotic region may become more apparent after enhancement, it is acceptable to employ the ROI placement method in this study, given prior reports demonstrating its efficacy and reliability in measuring the entire tumor, including internal necrosis [16].

The slope *K* of the Hounsfield Unit (HU) spectral curve, which reflects the CT value of 40–140 keV energy, was calculated using the following equation [17]:

$${{{\rm{slope}}}\;K\,=\,\frac{{{\rm{mean}}}\; {{\rm{CT}}}\; {{\rm{value}}}\;\left(40\,{{\rm{keV}}}\right)\,-\,{{\rm{mean}}}\; {{\rm{CT}}}\; {{\rm{value}}}\;\left(140\,{{\rm{keV}}}\right)}{140\,-\,40}}.$$

Subsequently, the two observers used the GSI viewer to assess the MD images. MD analysis quantified blood, fat, and effective *Z* (Eff-*Z*). The liver lesions’ Eff-*Z* minimum (min), mean, and maximum (max) values were calculated. Also determined was the difference between the maximum and minimum values (Eff-*Z* dif). Analyzing MD images of blood–water pairs determined the liver lesions’ blood concentration [mg/cc]. The multi-material decomposition (MMD) method, specifically the GE HealthCare GSI liver fat algorithm, quantified liver lesions’ fat fractions as percentages.

### The establishment of predictive models

All the calculated quantitative imaging data were used to create PMs for differentiating benign from malignant liver lesions. This was performed using multivariate logistic regression. To construct accurate PMs to distinguish between benign and malignant lesions, it is important to have equal sample sizes in both groups [18]. Therefore, the group with a small sample size was oversampled to increase the sample size. To reduce the risk of over-fitting in standard over-sampling, we employed the synthetic minority over-sampling technique (SMOTE) to address the issue while working with imbalanced datasets [19].

The process of constructing PMs is outlined below.

Step 1: Initially, the maximum number of explanatory variables to be incorporated in the PMs was established by dividing the number of cases in the minority group by 10 in both the benign and malignant lesion groups. The concept of requiring a minimum of 10 events per variable is a widely acknowledged principle for fitting logistic regression models and is supported by several studies and guidelines [20].

Step 2: The minority group was oversampled using SMOTE to achieve a sample size that is approximately equivalent to that of the majority group.

Step 3: Univariate analysis was conducted for all explanatory variables to distinguish between benign and malignant lesions. The *p*-values for each explanatory variable were obtained using a likelihood ratio test.

Step 4: A receiver operating characteristic (ROC) analysis was conducted for each explanatory variable to distinguish between benign and malignant lesions, and the area under the curve (AUC) was obtained.

Step 5: Variables that had both statistically significant *p*-values in the univariate analysis and high AUCs were given a preference. Representative explanatory variables were chosen from the VMI and MD images to exclude variables that were similar in nature.

Step 6: Based on the chosen explanatory variables, we included variables up to the maximum number determined in Step 1 and conducted multivariate logistic regression to assess the ability of the PMs to distinguish between benign and malignant lesions. The *p*-values for each variable were obtained from the parameter estimates. We excluded PMs that included explanatory variables that did not demonstrate statistical significance.

Step 7: Finally, PMs were established, wherein every explanatory variable exhibited statistical significance.

### Validation of predictive models

Two additional abdominal radiologists, Observers 3 and 4, with 6 years and 4 years of abdominal imaging experience, respectively, verified the PMs’ discriminative abilities of the validation group. In the validation cohort, Observers 3 and 4 calculated identical quantitative values. All the calculated quantitative imaging data were used to test the PMs’ ability to differentiate between benign and malignant liver lesions.

### Statistical analysis

The Shapiro–Wilk test showed no normal distribution of quantitative values. Therefore, non-parametric tests were performed. The Mann–Whitney *U*-test was used to calculate the significant difference in age between the derivation and validation cohorts. Fisher’s exact test was used to calculate significant differences in sex proportions. The two observers’ quantitative results correlations were assessed using Spearman’s rank correlation coefficients [21]. CT values at 40 keV, 70 keV, and 140 keV, slope *K*, Eff-*Z* min, mean, Max, dif, blood, and fat were compared between HH, HCC, MLC, and ICC using the Kruskal–Wallis and post hoc Steel–Dwass tests. We constructed PMs to distinguish between benign and malignant lesions by oversampling a smaller population using SMOTE to balance the sample sizes of the two groups in the derivation cohort. We used WEKA 3.8.6 software (Waikato Environment for Knowledge Analysis) with nearestNeighbors set to 5 and randomSeed set to 1, both default values. After univariate analysis, five multivariate logistic regression models were confirmed as the predictors of benign and malignant liver lesions. ROC analysis was used to examine the AUC of each model to determine its ability to distinguish between benign and malignant liver lesions. The appropriate cut-off value for each model was calculated using the Youden index. We used Observers 3 and 4 parameters to validate the models. Sensitivity, specificity, and accuracy were calculated, classifying values below the cut-off as benign and those above as malignant. Fisher’s exact test was also used to examine differences in diagnostic performance between groups with and without liver cirrhosis. Statistical analyses were performed using JMP Pro 17 (SAS Institute Inc.). *p*-values under 0.05 were statistically significant.

## Results

### Demographic characteristics of the patients

One hundred and 57 patients with liver lesions underwent non-CECT and multiphasic CT using a DECT scanner. Of these, 36 patients were eliminated from the study because they met the exclusion criteria. Patients with liver lesions previously treated with transarterial chemoembolization (TACE) or radiofrequency ablation (RFA) were not excluded. Of the 58 patients diagnosed with HCC, 10 received prior treatment with TACE, seven received prior treatment with RFA, and three received both TACE and RFA. However, it is important to note that in the patients included in this study, the lesions observed were not local recurrences at the treated site, but rather emerging lesions in a separate location. Our study did not examine only the partially treated lesions. Consequently, these patients were included in this study. The final research population included 121 patients. There were no statistically significant differences in age and sex proportions between the derivation and validation cohorts (*p* = 0.25, 0.23, respectively). The derivation cohort included 25 HH (25.8%), 47 HCC (48.5%), 17 MLC (17.5%), and 8 ICC cases (8.2%). The validation cohort had 7 HH (29.2%), 11 HCC (45.8%), 3 MLC (12.5%), and 3 ICC cases (12.5%). The diagnostic criteria for each liver lesion are summarized in Table 1. The diagnosis of all 32 HHs, 27 of 58 HCCs, 3 of 20 MLCs, and 1 of 11 ICCs of total liver lesions was confirmed only via CT and MR imaging (some are also evaluated by ultrasonography). A histopathological diagnosis was made for the remaining liver lesions. In summary, all 32 benign tumors (HH) were diagnosed by imaging modalities. Out of the 89 malignant tumors (HCC, MLC, and ICC), 58 were diagnosed through histological examination, while the remaining 31 were diagnosed through imaging methods. Table 1 and Fig. 1 summarize the patient data from the study. Twelve patients with HH, diagnosed solely through imaging, showed no enlargement on the follow-up imaging. Of the 27 patients with HCC, 24 were monitored by imaging. Among these, 10 showed lesion enlargement and 10 underwent TACE, demonstrating good lipiodol retention. The remaining four received RFA and showed no viable lesions on follow-up. All three patients with MLC showed progression on follow-up imaging. One patient with ICC did not undergo further imaging because of age and personal choice.

**Table 1:** Demographic information of the patient

	Total	Derivation cohort	Validation cohort
No. of patients	121	97	24
Mean age (y) (range)	67.36 ± 13.83 (29–93)	66.56 ± 14.31 (29–93)	70.58 ± 11.35 (47–92)
Sex			
No. of men	80 (66.1%)	67 (69.1%)	13 (54.2%)
No. of women	41 (33.9%)	30 (30.9%)	11 (45.8%)
Tumor size (mm) (range)	32.81 ± 21.65 (10–140)	31.68 ± 20.72 (10–140)	37.38 ± 25.06 (13–113)
Tumor type			
HH	32 (26.5%)	25 (25.8%)	7 (29.2%)
Diagnosis criteria			
CECT and US	20 (62.5%)	16 (64.0%)	4 (57.1%)
CECT only	8 (25.0%)	6 (24.0%)	2 (28.6%)
CECT and US and MRI	4 (12.5%)	3 (12.0%)	1 (14.3%)
HCC	58 (47.9%)	47 (48.5%)	11 (45.8%)
Diagnosis criteria			
Surgery and histopathology	31 (53.4%)	27 (57.4%)	4 (36.4%)
CECT and EOB-MRI	12 (20.7%)	10 (21.3%)	2 (18.2%)
CECT only	11 (19.0%)	7 (14.9%)	4 (36.4%)
CECT and EOB-MRI and CEUS	4 (6.9%)	3 (6.4%)	1 (9.1%)
MLC	20 (16.5%)	17 (17.5%)	3 (12.5%)
Diagnosis criteria			
Surgery and histopathology	16 (80.0%)	13 (76.5%)	3 (100%)
CECT only	2 (10.0%)	2 (11.8%)	0 (0%)
CECT and EOB-MRI	1 (5.0%)	1 (5.9%)	0 (0%)
Needle biopsy	1 (5.0%)	1 (5.9%)	0 (0%)
Primary site			
Colorectal cancer	14 (70.0%)	11 (64.7%)	3 (100%)
GIST	3 (15.0%)	3 (17.6%)	0 (0%)
Esophageal cancer	1 (5.0%)	1 (5.9%)	0 (0%)
Gastric cancer	1 (5.0%)	1 (5.9%)	0 (0%)
Endometrial cancer	1 (5.0%)	1 (5.9%)	0 (0%)
ICC	11 (9.1%)	8 (8.2%)	3 (12.5%)
Diagnosis criteria			
Surgery and histopathology	8 (3: cHCC-ICC) (72.7%)	5 (1: cHCC-ICC) (62.5%)	3 (2: cHCC-ICC) (100%)
Needle biopsy	2 (18.2%)	2 (25.0%)	0 (0%)
CECT only	1 (9.1%)	1 (12.5%)	0 (0%)

The numbers in parentheses represent the proportion of the total

*HH* hepatic hemangioma, *HCC* hepatocellular carcinoma, *MLC* metastatic liver cancer, *ICC* intrahepatic cholangiocellular carcinoma, *CECT* contrast-enhanced CT, *US* ultrasonography, *CEUS* contrast-enhanced ultrasonography, *cHCC-ICC* combined type hepatocellular carcinoma and intrahepatic cholangiocellular carcinoma

In addition, several patients with liver cirrhosis were included in our study. Specifically, one of the 32 patients with HH (3.1%) and 14 of the 58 patients with HCC (24.1%) had cirrhosis in the background liver. Seven of the 32 patients with HH (21.9%), 9 of the 58 patients with HCC (15.5%), 10 of the 20 patients with MLC (50.0%), and 0 of the 11 patients with ICC (0%) had multiple lesions. The diameters of the smallest lesions among the multiple lesions measured ranged from 8–19 mm for HHs, 7–25 mm for HCCs, and 5–25 mm for MLCs. Non-CECT allowed for the detection of all single lesions. However, several small lesions among the multiple lesions could not be detected on non-CECT. These lesions had diameters ranging from 8 mm to 10 mm in HH, 7 mm to 12 mm in HCC, and 5 mm to 10 mm in MLC. In our cohort, there were no cases in which different types of lesions were simultaneously present.

### Quantitative data results in the derivation cohort

Table 2 shows the median and interquartile range for 40 keV, 70 keV, and 140 keV CT values, and slope *K*, as well as the minimum, mean, maximum, and difference values for Eff-*Z*, blood quantification, and fat quantification. Observers 1 and 2 calculated these parameters. Spearman’s rank correlation coefficients (ρ) between observers for each parameter ranged from 0.74 to 0.99, with *p*-values < 0.0001. These coefficients show strong to very strong correlations. Table 2 summarizes both observers’ quantitative parameters.

**Table 2:** Parameter measurements and correlation results from four observers

Derivation cohort, (*n* = 97)	Observer 1		Observer 2		Average		Spearman’s coefficient	
	Median	IQR	Median	IQR	Median	IQR	ρ	*p*-value
CT value 40 keV	55.15	42.66–62.35	56.54	49.98–63.67	56.10	50.24–62.90	0.95	< 0.0001*
CT value 70 keV	45.74	41.08–49.37	45.85	40.82–50.22	45.89	41.06–49.79	0.88	< 0.0001*
CT value 140 keV	41.21	35.83–44.86	40.90	35.91–44.53	40.88	35.87–44.79	0.98	< 0.0001*
Slope *K*	0.16	0.085–0.23	0.17	0.094–0.23	0.16	0.096–0.23	0.96	< 0.0001*
Eff-*Z* min	7.31	7.17–7.38	7.31	7.24–7.38	7.31	7.21–7.38	0.74	< 0.0001*
Eff-*Z* mean	7.72	7.65–7.77	7.72	7.66–7.77	7.72	7.65–7.77	0.96	< 0.0001*
Eff-*Z* max	8.06	7.98–8.22	8.06	7.98–8.17	8.06	7.98–8.20	0.93	< 0.0001*
Eff-*Z* dif	0.74	0.6–0.99	0.79	0.6–0.96	0.77	0.64–0.98	0.88	< 0.0001*
Blood (water)	695.24	463.62–934.03	676.22	501.50–943.67	712.18	485.66–933.30	0.98	< 0.0001*
Fat (MMD)	10.74	7.08–15.21	10.48	6.66–15.45	10.62	6.99–15.38	0.99	< 0.0001*

Validation cohort, (*n* = 24)	Observer 3		Observer 4		Average		Spearman’s coefficient	
	Median	IQR	Median	IQR	Median	IQR	ρ	*p*-value
CT value 40 keV	54.65	46.26–66.30	55.27	46.99–66.64	N/A	N/A	0.98	< 0.0001*
CT value 70 keV	43.79	39.75–48.40	43.46	39.53–48.57	N/A	N/A	0.95	< 0.0001*
CT value 140 keV	39.81	34.46–43.28	39.56	33.95–44.92	N/A	N/A	0.96	< 0.0001*
Slope *K*	0.23	0.091–0.23	0.17	0.084–0.23	N/A	N/A	0.98	< 0.0001*
Eff-*Z* min	7.31	7.19–7.46	7.31	7.19–7.38	N/A	N/A	0.86	< 0.0001*
Eff-*Z* mean	7.72	7.65–7.78	7.70	7.64–7.75	N/A	N/A	0.89	< 0.0001*
Eff-*Z* max	8.09	7.99–8.14	8.09	7.98–8.16	N/A	N/A	0.88	< 0.0001*
Eff-*Z* dif	0.69	0.6–1.0075	0.73	0.6–0.94	N/A	N/A	0.92	< 0.0001*
Blood (water)	671.83	418.13–967.26	674.17	460.45–964.14	N/A	N/A	0.95	< 0.0001*
Fat (MMD)	11.86	8.06–16.13	11.66	7.55–15.58	N/A	N/A	0.99	< 0.0001*

*IQR* interquartile range, *ρ* Spearman’s rank correlation coefficient, *Eff-Z min* minimum value of effective-*Z*, *Eff-Z mean* mean value of effective-*Z*, *Eff-Z max* maximum value of effective-*Z*, *Eff-Z dif* difference between maximum and minimum effective-*Z*, *MMD* multi-material decomposition

* Asterisks indicate statistically significant differences

**Fig. 3:**
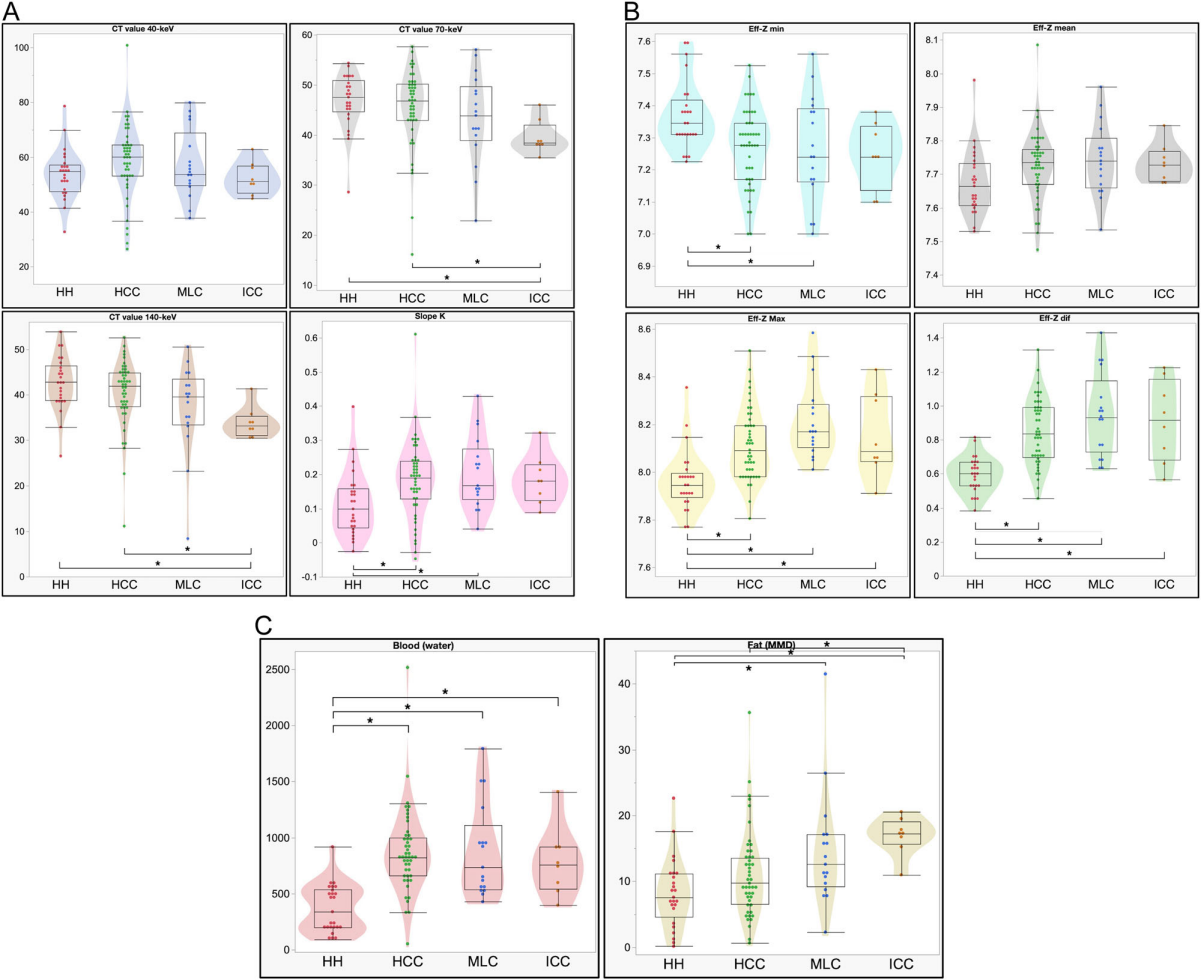
Box and violin plots displaying the individual parameters obtained from nCE-DECT in liver lesions. The median value is denoted by the center line of the box diagram, the interquartile range is represented by the box, and the range of values is denoted by the whiskers. In the context of a violin plot, the approximate frequency of data points in each region is represented by the width of each curve. The dots symbolize the individual observer’s assessment. Asterisks indicate a statistically significant difference. **A** Box plots of virtual monoenergetic images (VMI) displaying the results of CT values at 40 keV, 70 keV, and 140 keV and slope *K*. **B** Box plots representing the minimum and maximum Eff-*Z* values (Eff-*Z* min, mean, and max, respectively), as well as the difference between the two (Eff-*Z* dif). **C** Box plots illustrate the quantification of blood using water as the material pair and fat via mutimaterial decomposition (MMD), respectively

Table 3 and Fig. 3 show multi-parameter comparisons between HH, HCC, MLC, and ICC. None of the four groups had significantly different CT values at 40 keV. However, the CT values at 70 keV and 140 keV were significantly different in the HH and ICC groups (*p* = 0.0062 and 0.0054, respectively) and the HCC and ICC groups (*p* = 0.024 and 0.016, respectively). The slope *K* of HCC and MLC was substantially higher than that of HH (*p* = 0.018 and 0.027). Eff-*Z* min showed a statistically significant difference only between HH and HCC (*p* = 0.017). No significant differences were observed in the Eff-Z-mean between the groups. For HCC, MLC, and ICC, Eff-*Z* Max had significantly higher values than HH (*p* = 0.0001, < 0.0001, and 0.021, respectively). HCC, MLC, and ICC had significantly higher Eff-*Z* dif values than HH (*p* < 0.0001, *p* < 0.0001, *p* = 0.0087, respectively). HCC, MLC, and ICC had significantly higher blood (water) values than HH (*p* < 0.0001, 0.0001, and 0.0083, respectively). The MMD method showed significant fat differences between HH and MLC, HH and ICC, and HCC and ICC (*p* = 0.016, 0.0035, and 0.0016, respectively) (Table 3 and Fig. 3).

**Table 3:** Results for multiple parameters for the derivation cohort

		CT value 40 keV	CT value 70 keV	CT value 140 keV	Slope *K*	Eff-*Z* min
(A) HH	Median	54.82	47.54	42.79	0.099	7.35
	IQR	47.53–57.15	44.65–50.92	38.75–46.44	0.043–0.16	7.31–7.42
(B) HCC	Median	60.10	46.80	41.95	0.19	7.28
	IQR	53.06–64.44	42.95–50.15	37.47–44.88	0.13–0.24	7.17–7.35
(C) MLC	Median	53.65	43.88	39.55	0.17	7.24
	IQR	49.63–68.87	38.97–49.70	33.41–43.43	0.084–0.27	7.16–7.39
(D) ICC	Median	51.05	38.46	33.23	0.18	7.24
	IQR	46.95–56.79	38.01–42.03	31.10–35.34	0.12–0.23	7.14–7.34
*p*-value between four		0.072	0.013*	0.0033*	0.0086*	0.016*
(A) vs (B)		0.086	0.96	0.63	0.018*	0.017*
(A) vs (C)		0.90	0.56	0.20	0.027*	0.19
(A) vs (D)		0.96	0.0062*	0.0054*	0.13	0.088
(B) vs (C)		0.90	0.68	0.53	0.97	1.00
(B) vs (D)		0.22	0.024*	0.016*	1.00	0.92
(C) vs (D)		0.83	0.34	0.31	0.99	0.99

		Eff-*Z* mean	Eff-*Z* max	Eff-*Z* dif	Blood (water)	Fat (MMD)
(A) HH	Median	7.67	7.95	0.60	339.44	7.58
	IQR	7.61–7.73	7.89–8.00	0.53–0.67	198.93–536.79	4.58–11.13
(B) HCC	Median	7.74	8.09	0.84	818.56	9.71
	IQR	7.67–7.78	7.98–8.20	0.70–0.99	658.69–996.29	6.53–13.52
(C) MLC	Median	7.74	8.17	0.93	735.92	12.59
	IQR	7.66–7.81	8.10–8.29	0.73–1.15	539.35–1111.14	9.20–17.13
(D) ICC	Median	7.73	8.09	0.92	756.82	17.27
	IQR	7.68–7.77	8.05–8.32	0.68–1.16	544.24–916.75	15.62–19.09
*p*-value between four		0.037*	< 0.0001*	< 0.0001*	< 0.0001*	0.0003*
(A) vs (B)		0.062	0.0001*	< 0.0001*	< 0.0001*	0.25
(A) vs (C)		0.099	< 0.0001*	< 0.0001*	< 0.0001*	0.016*
(A) vs (D)		0.20	0.021*	0.0087*	0.0083*	0.0035*
(B) vs (C)		0.96	0.12	0.60	0.97	0.28
(B) vs (D)		1.00	0.94	0.90	0.83	0.016*
(C) vs (D)		1.00	0.82	0.97	0.97	0.31

*Eff-Z min* minimum value of effective-Z, *Eff-Z mean* mean value of effective-*Z*, *Eff-Z max* maximum value of effective-*Z*, *Eff-Z dif* difference between maximum and minimum effective-*Z*, *MMD* multi-material decomposition, *HH* hepatic hemangioma, *HCC* hepatocellular carcinoma, *MLC* metastatic liver cancer, *ICC* intrahepatic cholangiocellular carcinoma, *IQR* interquartile range

* Asterisks indicate statistically significant differences

### Results of the predictive model's reconstruction

The following are the results that correspond to Steps 1 through 7 of the “Methods”.

Result 1: The minority group (benign lesions) comprised 25 cases. Therefore, the maximum number of explanatory variables to be incorporated into PMs was set to 3 (25/10 = 2.5).

Result 2: The utilization of SMOTE for oversampling led to the creation of a balanced dataset consisting of 75 cases in the minority group (benign lesions) and 72 cases in the majority group (malignant lesions).

Result 3: Univariate analysis revealed that all variables were statistically significant: CT values at 40 keV, 70 keV, and 140 keV; slope *K*; Eff-*Z* min; Eff-*Z* mean; Eff-*Z* Max; Eff-*Z* dif; blood; and fat (all *p* < 0.05). Table 4 shows the odds ratios and *p*-values for each variable.

**Table 4:** Results from both univariate and multivariate analyses

Variable	Odds ratio	Parameter estimates	*p*-value	AUC
Univariate analysis				
CT value 40 keV	1.04 (1.01, 1.07)	N/A	0.016*	0.62
CT value 70 keV	0.93 (0.88, 0.99)	N/A	0.010*	0.61
CT value 140 keV	0.92 (0.86, 0.97)	N/A	0.0010*	0.63
Slope *K*	9733.84 (147.71, 941434.2)	N/A	< 0.0001*	0.73
Eff-*Z* min	0.00056 (1.97e-5, 0.016)	N/A	< 0.0001*	0.71
Eff-*Z* mean	520.94 (11.07, 24,505.49)	N/A	0.0006*	0.69
Eff-*Z* max	14,173.07 (470.91, 7,426,566.5)	N/A	< 0.0001*	0.83
Eff-*Z* dif	174,452 (2983.64, 10,200,135)	N/A	< 0.0001*	0.88
Blood	1.01 (1.01, 1.01)	N/A	< 0.0001*	0.91
Fat	1.15 (1.07, 1.23)	N/A	< 0.0001*	0.70
Multivariate logistic regression analysis				
Model 1				
Slope *K*	8.58e-36 (0, 1.06e-19)	−80.74 (−123.70, −48.60)	< 0.0001*	0.98
Blood	1.032 (1.019, 1.044)	0.032 (0.021, 0.045)	< 0.0001*	
Intercept	N/A	−5.99 (−8.59, −4.080)	< 0.0001*	
logit 1 = −80.74 × slope *K* + 0.032 × blood −5.99				
Model 2				
Slope *K*	1700.02 (21.02, 137,503.6)	7.44 (3.23, 12.057)	0.0009*	0.76
Fat	1.10 (1.00, 1.21)	0.10 (0.032, 0.18)	0.0060*	
Intercept	N/A	−2.15 (−3.15, −1.27)	< 0.0001*	
logit 2 = 7.44 × slope *K* + 0.10 × fat −2.15				
Model 3				
Eff-*Z* dif	7412.54 (101.66, 540,482.9)	8.91 (5.014, 13.67)	< 0.0001*	0.95
Blood	1.0072 (1.0041, 1.010)	0.0071 (0.0044, 0.011)	< 0.0001*	
Intercept	N/A	−10.33 (−14.54, −7.14)	< 0.0001*	
logit 3 = 8.91 × Eff-*Z* dif + 0.0071 × blood −10.33				
Model 4				
Slope *K*	9.87e-43 (0, 1.80e-22)	−96.72 (−152.16, −57.051)	< 0.0001*	0.98
Blood	1.035 (1.020, 1.050)	0.034 (0.022, 0.052)	< 0.0001*	
Fat	1.21 (1.044, 1.41)	0.19 (0.049, 0.35)	0.011*	
Intercept	N/A	−7.53 (−11.030, −5.054)	< 0.0001*	
logit 4 = −96.72 × Slope *K* + 0.034 × blood + 0.19 × fat −7.53				
Model 5				
Slope *K*	1.86e-41 (0, 1.66e-18)	−93.78 (−157.48, −49.98)	0.0005*	0.99
Eff-Z dif	857,743.4 (221.12, 3.33e + 9)	13.66 (6.50, 23.60)	0.0012*	
Blood	1.034 (1.017, 1.052)	0.034 (0.020, 0.055)	< 0.0001*	
Intercept	N/A	−15.034 (−23.96, −9.16)	< 0.0001*	
logit 5 = −93.78 × slope *K* + 13.66 × Eff-Z dif + 0.034 × blood −15.034				

The numbers in parentheses are 95% confidence intervals

*Eff-Z min* minimum value of effective-Z, *Eff-Z mean* mean value of effective-Z, *Eff-Z max* maximum value of effective-Z, *Eff-Z dif* difference between maximum and minimum effective-*Z*

* Asterisks indicate statistically significant differences

Result 4: A summary of the AUCs for each explanatory variable is provided in Table 4. Among the VMI variables, slope *K* exhibited the highest AUC value, whereas blood demonstrated the highest AUC value among the MD image variables.

Result 5: Slope *K* (*p* < 0.0001, AUC = 0.73) was chosen as the representative of the VMI variable, while blood (*p* < 0.0001, AUC = 0.91) was selected as the representative of the MD image variable. Of the Eff-*Z* variables, we chose Eff-*Z* dif (*p* < 0.0001, AUC = 0.88), which had the highest AUC. Fat had a relatively low AUC of 0.70. However, because of its significant role in tumor differentiation, we selected it as an explanatory variable.

Result 6: Of the four explanatory variables, two or three were chosen to create candidate PMs. Of the ten combinations, the combinations of slope *K* and Eff-*Z* dif (*p* = 0.27, *p* < 0.0001); Eff-Z dif and fat (*p* < 0.0001, 0.36); blood and fat (*p* < 0.0001, 0.097); slope *K*, Eff-*Z* dif, and fat (*p* = 0.39, *p* < 0.0001, 0.55); and Eff-*Z* dif, blood, and fat (*p* = 0.0004, *p* < 0.0001, 0.74) were excluded because they contained variables that were not statistically significant.

Result 7: Five models that were finally conducted as PMs were as follows: Model 1 (slope *K* and blood, both *p* < 0.0001), Model 2 (slope *K* and fat, *p* = 0.0009, 0.0060), Model 3 (Eff-*Z* dif and blood, both *p* < 0.0001), Model 4 (slope *K*, blood, and fat, *p* < 0.0001, < 0.0001, 0.011), and Model 5 (slope *K*, Eff-*Z* dif, and blood, *p* = 0.0005, 0.0012, < 0.0001). Table 4 summarizes the odds ratios and parameter estimates, as well as their 95% confidence intervals, for each model parameter.

The following were the prediction equations for the five models:

$${{\bullet }}{{\rm{Model}}}\,1\,=\, -80.74\,\times \,{{\rm{slope}}}\,K\,+\,0.032\, \times \,{{\rm{blood}}}\,{-}\,5.99$$

$$\bullet {{\rm{Model}}}\;2\,=\,7.44\, \times \,{{\rm{slope}}}\; {K}\,+\,0.10\, \times \,{{\rm{fat}}}\,{-}\,2.15$$

$$\bullet {{\rm{Model}}}\;3\,=\,8.91\, \times \,{{\rm{Eff}}}{\mbox{-}}{Z}\;{{\rm{diff}}}\,+\,0.0071\, \times \,{{\rm{blood}}}\,{-}\,10.33$$

$$\bullet {{\rm{Model}}}\;4\, = 	 \,{-}96.72\, \times \,{{\rm{slope}}}\;{K}\,+\,0.034\, \times \,{{\rm{blood}}}\, \\ 	 +\,0.19\, \times \,{{\rm{fat}}}\,{-}\,7.53$$

$$\bullet {{\rm{Model}}}\;5\, = 	 \,{-}93.78\, \times \,{{\rm{slope}}}\;{K}\,+\,13.66\, \times \,{{\rm{Eff}}}{\mbox{-}}{Z}\;{{\rm{diff}}}\, \\ 	 +\,0.034\, \times \,{{\rm{blood}}}\,{-}\,15.034$$

**Fig. 4:**
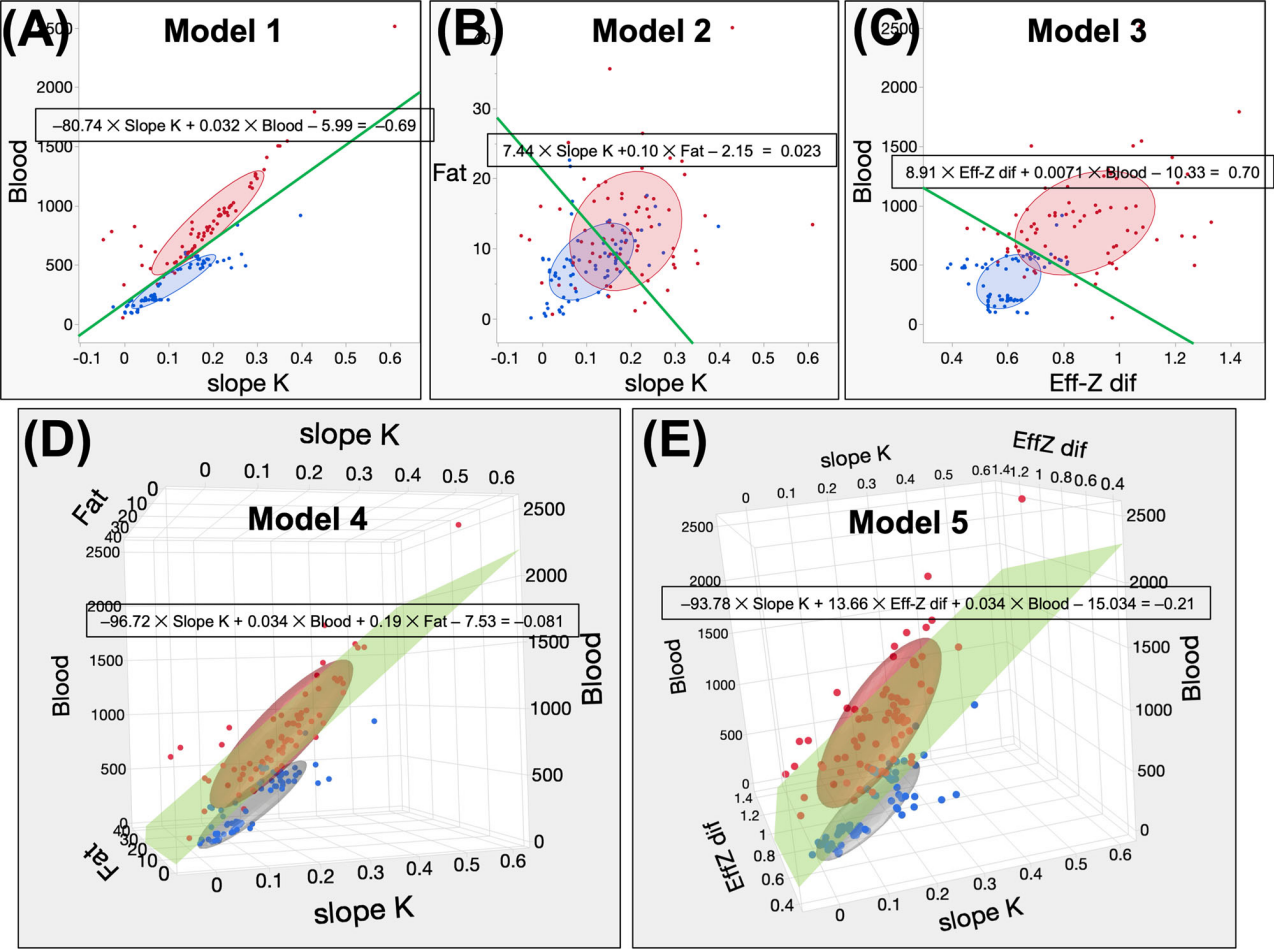
Scatter plots of five logistic regression models derived from the cohort of derivation. **A**–**C** Scattered 2D images of Models 1 through 3. Red dots denote malignant liver lesions, while blue dots represent benign liver lesions. The elliptical distribution of benign lesions (α = 0.5) is represented by the blue ellipse, while the elliptical distribution of malignant lesions (α = 0.5) is illustrated by the red ellipse. **A** Scatter plot depicting the data points of Model 1. The *x*-axis represents the variable “slope *K*,” while the *y*-axis represents the variable “blood.” The green line represents the regression equation for a value of 1.33. **B** Graphical representation of the data points in Model 2 using a scatter plot. The *x*-axis represents the variable “slope *K*,” while the *y*-axis represents the variable “fat.” The green line represents the regression equation’s value of 0.76. **C** Graph depicting the distribution of data points for Model 3. The *x*-axis represents the difference in Eff-*Z* dif, whereas the *y*-axis represents blood. The green line shows the line where the regression equation is 0.16. **D**, **E** Scatter plots in three dimensions depict the data points of Models 4 and 5. The blue dots indicate the presence of benign liver lesions, while the red dots indicate the presence of malignant liver lesions. The blue ellipse represents the elliptical distribution of benign lesions with a shape parameter of α = 0.5, while the red ellipse represents the elliptical distribution of malignant lesions with the same shape parameter. **D** Three-dimensional scatter plot for Model 4. The *x*-axis represents the slope *K*, the *y*-axis represents fat, and the *z*-axis represents blood. The green plane represents the plane on which the regression equation has a value of 0.75. **E** Visualization of Model 5, represented as a three-dimensional scatter plot. The *x*-axis represents the slope *K*, the *y*-axis represents the Eff-*Z* dif, and the *z*-axis represents the blood. The green plane represents the plane on which the regression equation has a value of − 0.21

**Fig. 5:**
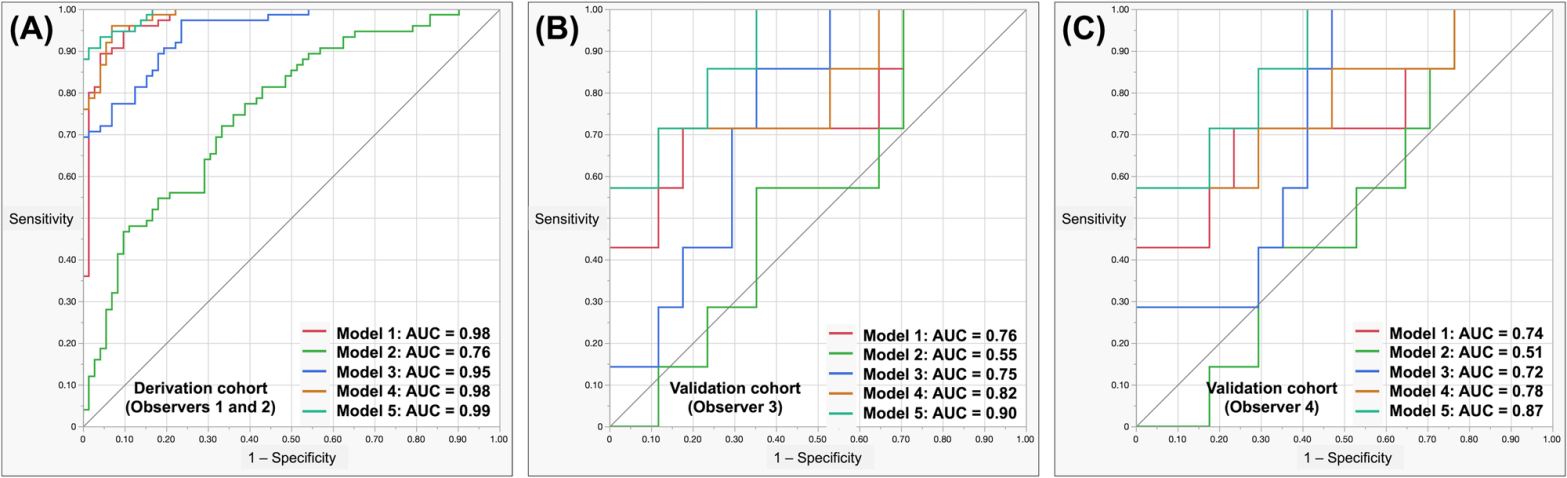
Performing ROC analysis to distinguish between benign and malignant lesions of the liver in both the derivation and validation cohorts. The ROC analyses of the five models are shown with their corresponding results. **A** A cohort is used for deriving or developing a model or analysis. **B** The validation cohort was assessed by Observer 3. **C** The validation cohort was observed by Observer 4

Table 4 also summarizes the AUCs of each model. Scatter plots of each model are shown in Fig. 4. The ROC analysis results for the five models in the derivation cohort are presented in Fig. 5A. Regardless of the presence or absence of liver cirrhosis, the diagnosing ability for all PMs in the derivation cohort did not differ significantly (Supplementary Table).

### Validation of the predictive models

**Fig. 6:**
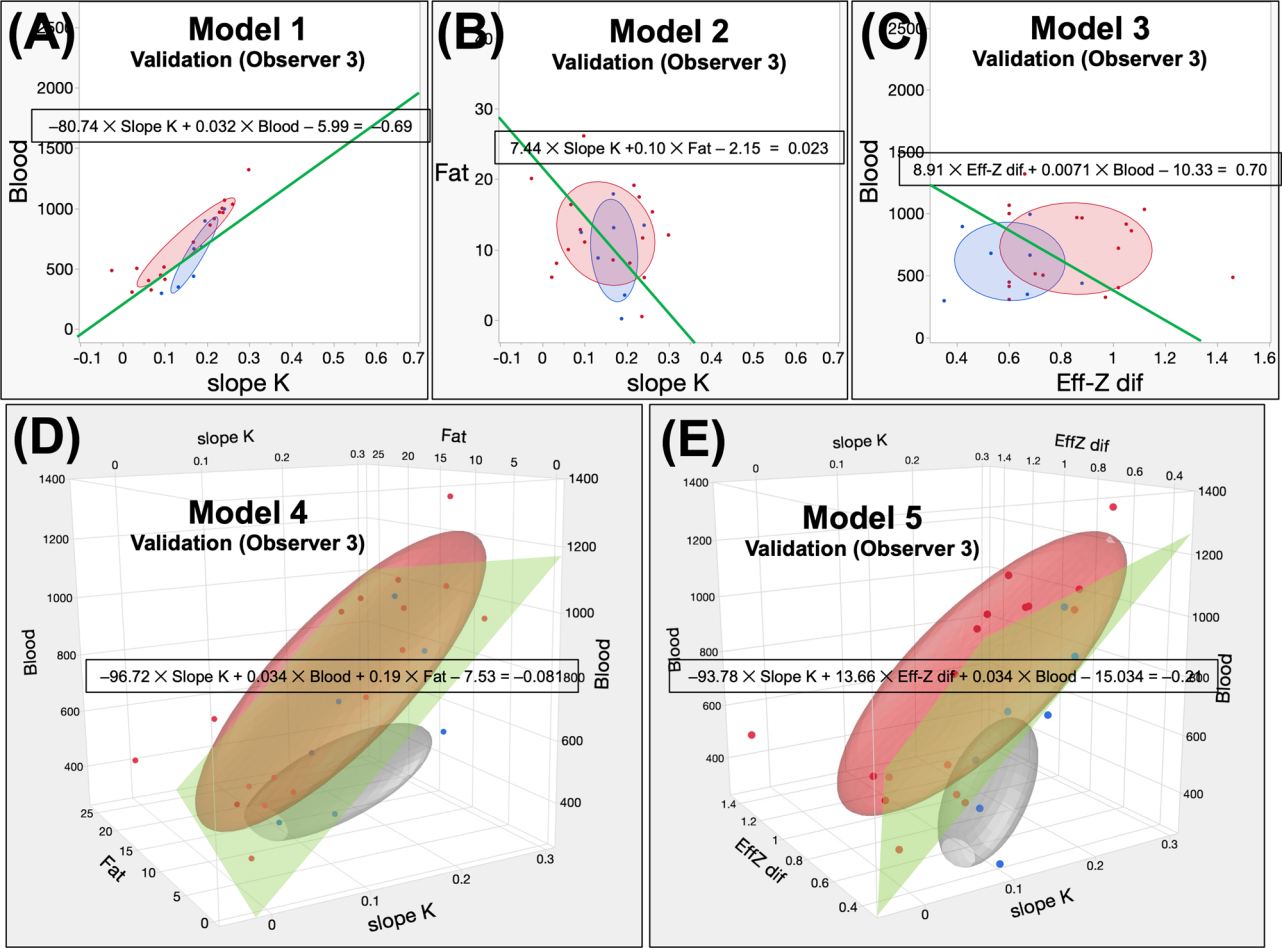

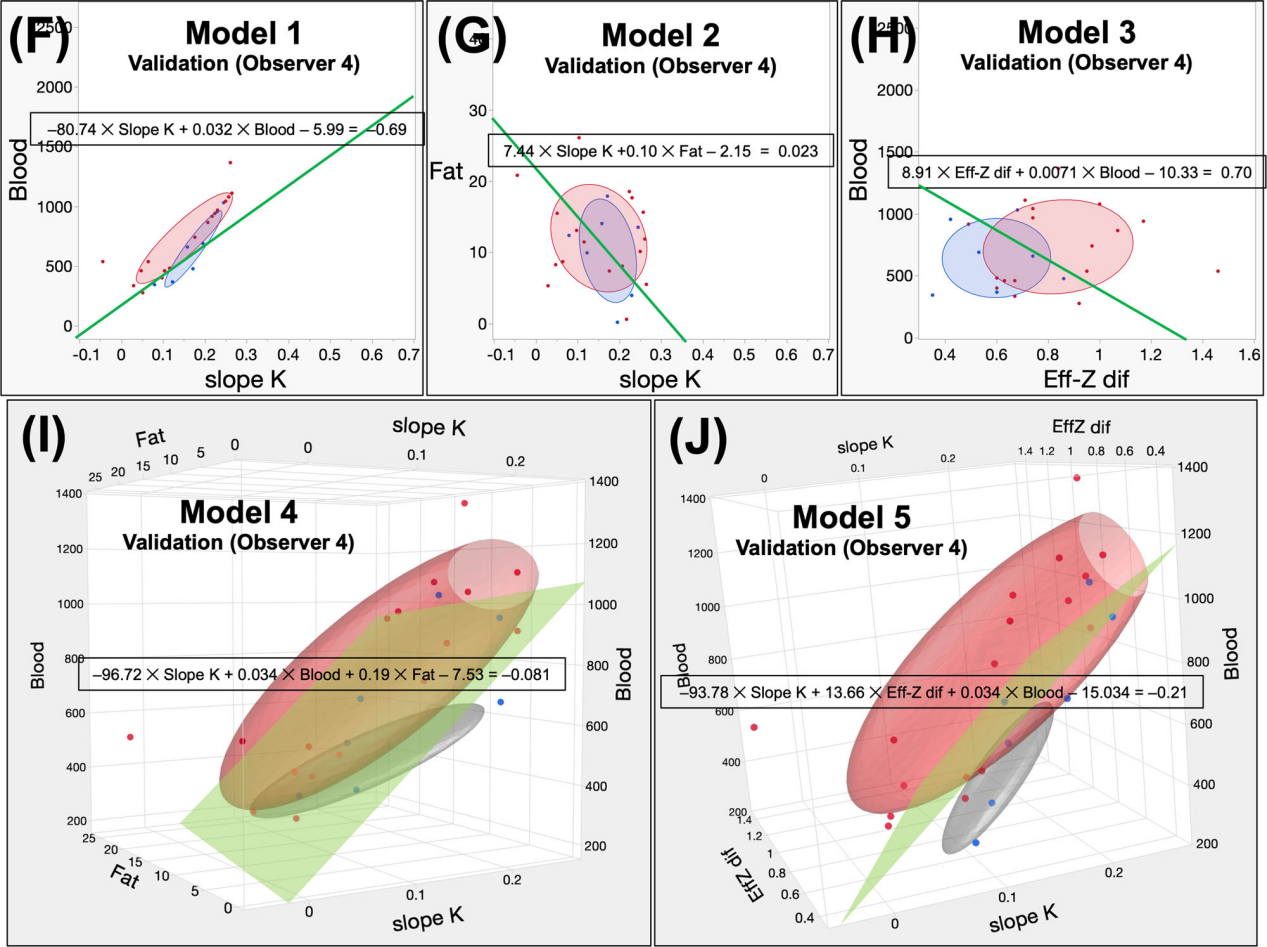
The results of the validation process in the validation cohort utilizing the five models of observers 3 and 4. **A**–**C**, **F**–**H** Scatter plots depicting Models 1 through 3 of observers 3 and 4. The blue dots represent benign liver lesions, while the red dots represent malignant liver lesions. The colored ovals represent an elliptical distribution with a value of α equal to 0.5. **D**, **E**, **I**, **J** Scatter plots in three dimensions depicting Models 4 and 5 of observers 3 and 4. The blue dots represent benign liver lesions, while the red dots represent malignant liver lesions. The colored ovals represent an elliptical distribution with a value of α equal to 0.5

Data from Observers 3 and 4 were used to apply Models 1–5 formulas in the validation cohort. The AUC, sensitivity, specificity, and accuracy of each model were calculated. According to Observer 3, the AUCs for Models 1, 2, 3, 4, and 5 were 0.76, 0.55, 0.75, 0.82, and 0.90, respectively. According to Observer 4, the AUC values for Models 1, 2, 3, 4, and 5 were 0.74, 0.51, 0.72, 0.78, and 0.87, respectively. Model 5 had the highest AUC for both observers, whereas Model 2 had the lowest. The appropriate cut-off values for each model were used to calculate the sensitivity and specificity (Table 5). When applying cut-offs, the sensitivity, specificity, and accuracy of Models 1 through 5 for Observers 3 and 4 are shown in Table 5. The *p-*values, which compare the AUCs for each model, are also presented in Table 5. Scatter plots from each model are shown in Fig. 6. The ROC analysis results for the five models in the validation cohort are summarized in Fig. 5B, C. Statistically, there was no significant difference in the diagnostic performance for all PMs in the validation cohort, regardless of the presence or absence of cirrhosis (Supplementary Table).

**Table 5:** Results of the ROCs analysis for each model

Model type	AUC	95% CI	Cut-off	Sensitivity (%)	Specificity (%)	Accuracy (%)	*p*-value			
Derivation cohort (Observers 1 and 2)										
(A) Model 1	0.98	0.94–0.99	− 0.69	95.8 (69/72)	88.0 (66/75)	91.8 (135/147)	(A) vs (B)	< 0.0001*	(B) vs (D)	< 0.0001*
(B) Model 2	0.76	0.67–0.82	0.023	65.3 (47/72)	72.0 (54/75)	68.7 (101/147)	(A) vs (C)	0.051	(B) vs (E)	< 0.0001*
(C) Model 3	0.95	0.90–0.97	0.7	76.4 (55/72)	97.3 (73/75)	87.1 (128/147)	(A) vs (D)	0.22	(C) vs (D)	0.010*
(D) Model 4	0.98	0.96–0.99	− 0.081	91.7 (66/72)	96.0 (72/75)	93.9 (138/147)	(A) vs (E)	0.12	(C) vs (E)	0.0016*
(E) Model 5	0.99	0.97–1.00	− 0.21	93.1 (67/72)	93.3 (70/75)	93.2 (137/147)	(B) vs (C)	< 0.0001*	(D) vs (E)	0.22
Validation cohort (Observer 3)										
(A) Model 1	0.76	0.45–0.93	− 0.69	88.2 (15/17)	42.9 (3/7)	75.0 (18/24)	(A) vs (B)	0.24	(B) vs (D)	0.083
(B) Model 2	0.55	0.31–0.77	0.023	47.1 (8/17)	57.1 (4/7)	50.0 (12/24)	(A) vs (C)	0.88	(B) vs (E)	0.0061*
(C) Model 3	0.75	0.51–0.90	0.7	64.7 (11/17)	85.7 (6/7)	70.8 (17/24)	(A) vs (D)	0.21	(C) vs (D)	0.52
(D) Model 4	0.82	0.50–0.95	− 0.081	94.1 (16/17)	57.1 (4/7)	83.3 (20/24)	(A) vs (E)	0.09	(C) vs (E)	0.021*
(E) Model 5	0.90	0.68–0.97	− 0.21	94.1 (16/17)	57.1 (4/7)	83.3 (20/24)	(B) vs (C)	0.084	(D) vs (E)	0.2
Validation cohort (Observer 4)										
(A) Model 1	0.74	0.43–0.91	− 0.69	88.2 (15/17)	42.9 (3/7)	75.0 (18/24)	(A) vs (B)	0.11	(B) vs (D)	0.028*
(B) Model 2	0.51	0.28–0.74	0.023	47.1 (8/17)	57.1 (4/7)	50.0 (12/24)	(A) vs (C)	0.88	(B) vs (E)	0.0014*
(C) Model 3	0.72	0.47–0.88	0.7	58.8 (10/17)	57.1 (4/7)	58.3 (14/24)	(A) vs (D)	0.35	(C) vs (D)	0.59
(D) Model 4	0.78	0.47–0.94	− 0.081	82.4 (14/17)	57.1 (4/7)	75.0 (18/24)	(A) vs (E)	0.1	(C) vs (E)	0.046*
(E) Model 5	0.87	0.64–0.96	− 0.21	82.4 (14/17)	57.1 (4/7)	75.0 (18/24)	(B) vs (C)	0.056	(D) vs (E)	0.18

*AUC* area under curve, *95% CI* 95% confidence interval

* Asterisks indicate statistically significant differences

## Discussion

This study involved the development of predictive models using nCE-DECT to distinguish between benign and malignant liver lesions in a derivation group of patients used for creating the model and the evaluation of its accuracy in a validation group of patients. Of the five models, the one built using slope *K*, Eff-Z dif, and blood exhibited the most excellent diagnostic performance (AUC = 0.90 and 0.87). Furthermore, these models could be adapted to liver lesions with or without liver cirrhosis. The clinical utility of distinguishing liver lesions using non-CECT is highly valuable, and prior research has not endeavored to differentiate liver lesions using nCE-DECT, thus making this finding novel.

Wang et al examined the application of quantitative DECT parameters to distinguish between liver abscesses and MLC [17]. Similar to our study, they compared CT values at 40 keV, 70 keV, and 140 keV, slope *K*, Eff-*Z*, blood (water), and fat (water) on nCE-DECT between abscesses and MLCs. Their report indicated that the values of slope *K*, blood (water), and fat (water) were greater in MLC than in abscesses. This aligns with our findings that malignant lesions were significantly higher than benign lesions. However, they also observed that the average Eff-Z was higher in MLC than in abscesses. In our study, we did not find a significant disparity in the average Eff-*Z* between benign and malignant lesions. Given the distinct characteristics of benign lesions, such as liver abscesses and HH, it is not possible to make a precise comparison. In their study, they also reported an AUC of 0.76 at a CT value of 40 keV to distinguish between abscess and MLC. However, in our study, the AUC for distinguishing between benign and malignant lesions was 0.62, which was lower than their findings [17]. This could also be attributed to the different types of liver lesions.

Slope *K* is a crucial parameter used for qualitative analysis of various tissues. The VMI’s CT values at different energy levels, usually 40 keV and 140 keV, are used for the calculation [17, 22]. The significance of calculating slope *K* in DECT lies in its capacity to offer valuable insights into the attenuation properties of diverse tissues, thereby facilitating the distinction and identification of various medical conditions. Consistent with prior research, the malignant lesions in this study exhibited a significantly greater slope *K* than the benign lesions [17, 22]. A key factor may be variability in cell density. Malignant lesions have a greater concentration of actively proliferating cells [23]. Changes in cellular composition may affect the interaction of cells with X-rays of varying energy, potentially leading to a change in their slope *K* value.

The effective atomic number of a material, denoted as Eff-*Z*, is directly proportional to its atomic number. Consequently, materials with a higher density have a correspondingly higher effective atomic number [24]. Prior studies have indicated that the mean Eff-*Z* was notably greater in malignant lesions than in benign lesions in the liver, kidney, ovary, breast, thyroid, and orbital regions [17, 24–28]. Conversely, our study found that the average Eff-*Z* was lower for benign lesions than for malignant lesions, although this difference did not reach statistical significance. There was a substantial disparity in the diversity of Eff-*Z* values (max minus min) between malignant and benign lesions. Because of the absence of prior research measuring the highest and lowest limits, no direct comparison can be drawn. However, this discovery potentially signifies variations in lesion heterogeneity. Past pathological investigations have shown that malignant liver lesions have more heterogeneous histological characteristics than benign lesions [29].

The quantified value of blood with water as the material pair suggests a small amount of bleeding in the lesions [17]. Regarding the quantitative blood values in this study, it is possible that malignant lesions exhibit higher values than benign HH because of the heterogeneity of their tissues and the presence of varying degrees of hemorrhage. In general, malignant liver lesions are more likely to cause spontaneous and substantial bleeding than benign lesions because of variables such as tumor invasion, necrosis, and disruption of vascular structures [30].

Concerning the measurement of fat percentage, prior research has indicated a certain level of correlation between the quantification of fat in liver lesions using MMD and the quantification of fat observed by MRI [31]. Although it is widely recognized that HCC and combined-type HCC-ICC contain fat [32, 33], this investigation revealed no significant difference in fat quantification between HCC and HH. The quantity of fat present in the HCCs exhibited substantial variation, indicating that most HCCs examined in this study were likely to have low-fat content. According to a previous study, MRI sometimes reveals intratumoral fat in colorectal liver metastases [34]. Based on this, the fat quantities observed in this study were greater than those in HH, as the predominant primary lesions in MLCs were colorectal cancers.

This study has several limitations. First, it was conducted retrospectively at a single institution, and the sample size was comparatively limited. Specifically, there are a limited number of validation cohorts, consisting of only 24 cases, and additional validation with a larger number of cases is required. Furthermore, not all cases were accompanied by histological diagnosis. However, in clinical practice, it is challenging to histologically diagnose all cases because benign lesions are typically not treated through surgery and not all HCCs undergo surgical resection because of the wide variety of treatment choices available. In this study, when a histological diagnosis was unattainable, the diagnosis relied on certain diagnostic standards in imaging. Most cases of malignant liver lesions diagnosed by imaging alone were followed up, and findings such as tumor growth or size changes responsive to treatment were confirmed. For HH, there were some cases in which follow-up imaging studies were not performed; however, if the lesions showed typical enhancement patterns on the dynamic study, the likelihood of misdiagnosis was considered extremely low. Moreover, of the 11 ICCs, three were classified as HCC–ICC combined. These lesions were included in the ICC group because of their characteristic features of ICC. Finally, we examined the diagnostic performance of each prediction model to distinguish between benign and malignant liver lesions, specifically in patients with or without cirrhosis. Nevertheless, not all instances were histopathologically confirmed as cirrhosis (F4), and a few were identified as cirrhosis solely based on clinical criteria.

In conclusion, this study focused on creating models that can effectively distinguish between benign and malignant liver lesions using nCE-DECT. The model, which included slope *K*, Eff-*Z* dif, and blood, demonstrated excellent diagnostic accuracy. The ability to distinguish between benign and malignant liver lesions without the need for contrast media is highly important in clinical settings, and our predictive model should be extensively used in clinical practice.

## Supplementary information

ELECTRONIC SUPPLEMENTARY MATERIAL

## Abbreviations

DECT: Dual-energy CT
Eff-*Z*: Effective *Z*
HH: Hepatic hemangioma
HCC: Hepatocellular carcinoma
ICC: Intrahepatic cholangiocellular carcinoma
MD: Material decomposition
MLC: Metastatic liver cancer
MMD: Multi-material decomposition
non-CECT: Non-contrast-enhanced CT
nCE-DECT: Non-contrast-enhanced DECT
VMI: Virtual monochromatic image
