# Supplementary material for: Prediction models for differentiating benign from malignant liver lesions based on multiparametric dual-energy non-contrast CT
Source: Eur Radiol. 2024 Aug 26;35(3):1361–77. doi: 10.1007/s00330-024-11024-8 (PMC11836082; doi:10.1007/s00330-024-11024-8)
Supplement: Supplementary file 1 — ELECTRONIC SUPPLEMENTARY MATERIAL [file 330_2024_11024_MOESM1_ESM.pdf]

Prediction models for differentiating benign from malignant liver lesions based on multiparametric dual-energy non-contrast CT

ELECTRONIC SUPPLEMENTARY MATERIAL

Supplementary Table: Comparison of diagnostic performance in each model with and without liver cirrhosis

	Models	Sensitivity (%)		*p*-value	Specificity (%)		*p*-value	Accuracy (%)		*p*-value
		LC (+)	LC (–)		LC (+)	LC (–)		LC (+)	LC (–)	
Derivation	**Model 1**	**100** (10/10)	**95.2** (59/62)	1.00	**100** (1/1)	**83.3** (20/24)	1.00	**100** (11/11)	**91.9** (79/86)	1.00
	**Model 2**	**40.0** (4/10)	**69.4** (43/62)	0.086	**100** (1/1)	**70.8** (17/24)	1.00	**45.4** (5/11)	**69.8** (60/86)	0.17
	**Model 3**	**60.0** (6/10)	**79.0** (49/62)	0.23	**100** (1/1)	**95.8** (23/24)	1.00	**63.6** (7/11)	**83.7** (72/86)	0.12
	**Model 4**	**100** (10/10)	**90.3** (56/62)	0.59	**100** (1/1)	**95.8** (23/24)	1.00	**100** (11/11)	**91.9** (79/86)	1.00
	**Model 5**	**100** (10/10)	**91.9** (57/62)	1.00	**100** (1/1)	**91.2** (22/24)	1.00	**100** (11/11)	**91.9** (79/86)	1.00
Validation (Observer 3)	**Model 1**	**100** (4/4)	**84.6** (11/13)	1.00	N/A	**42.9** (3/7)	N/A	N/A	**70.0** (14/20)	N/A
	**Model 2**	**50.0** (2/4)	**46.2** (6/13)	1.00	N/A	**57.1** (4/7)	N/A	N/A	**50.0** (10/20)	N/A
	**Model 3**	**50.0** (2/4)	**69.2** (9/13)	0.58	N/A	**85.7** (6/7)	N/A	N/A	**75.0** (15/20)	N/A
	**Model 4**	**100** (4/4)	**92.3** (12/13)	1.00	N/A	**57.1** (4/7)	N/A	N/A	**80.0** (16/20)	N/A
	**Model 5**	**100** (4/4)	**92.3** (12/13)	1.00	N/A	**57.1** (4/7)	N/A	N/A	**80.0** (16/20)	N/A
Validation (Observer 4)	**Model 1**	**75.0** (3/4)	**92.3** (12/13)	0.43	N/A	**42.9** (3/7)	N/A	N/A	**75.0** (15/20)	N/A
	**Model 2**	**50.0** (2/4)	**46.2** (6/13)	1.00	N/A	**57.1** (4/7)	N/A	N/A	**50.0** (10/20)	N/A
	**Model 3**	**50.0** (2/4)	**61.5** (8/13)	1.00	N/A	**57.1** (4/7)	N/A	N/A	**60.0** (12/20)	N/A
	**Model 4**	**75.0** (3/4)	**84.6** (11/13)	1.00	N/A	**57.1** (4/7)	N/A	N/A	**75.0** (15/20)	N/A
	**Model 5**	**75.0** (3/4)	**84.6** (11/13)	1.00	N/A	**57.1** (4/7)	N/A	N/A	**75.0** (15/20)	N/A

LC = liver cirrhosis. The two groups were compared using Fisher's exact test.

Eur Radiol (2024) Ota T, Onishi H, Fukui H, et al.
